# Lactobacillus Plantarum HFY15 Helps Prevent Retinoic Acid-Induced Secondary Osteoporosis in Wistar Rats

**DOI:** 10.1155/2020/2054389

**Published:** 2020-09-22

**Authors:** Xinhong Liu, Jiazhuang Zheng, Fang Li, Ruokun Yi, Jianfei Mu, Fang Tan, Xin Zhao

**Affiliations:** ^1^Chongqing Collaborative Innovation Center for Functional Food, Chongqing University of Education, Chongqing 400067, China; ^2^Chongqing Engineering Research Center of Functional Food, Chongqing University of Education, Chongqing 400067, China; ^3^Chongqing Engineering Laboratory for Research and Development of Functional Food, Chongqing University of Education, Chongqing 400067, China; ^4^College of Biological and Chemical Engineering, Chongqing University of Education, Chongqing 400067, China; ^5^Department of Spine Surgery, Suining Center Hospital, Chongqing Medical University, Suining, Sichuan 629000, China; ^6^Department of Public Health, Our Lady of Fatima University, Valenzuela 838, Philippines

## Abstract

A rat model of secondary osteoporosis was constructed using retinoic acid as an inducer, and the genes, proteins, and bone mass of the rats were analyzed. qPCR detection of the Wnt/*β*-catenin and OPG/RANK/RANKL signaling pathway-related gene expression levels showed that *Lactobacillus plantarum* HFY15 played a positive role in regulating both pathways. HFY15 significantly increased *β*-catenin, Lrp5, Lrp6, Wnt10b, OPG, RANKL, and Runx2 expression and downregulated DKK1, RANK, CTSK, TRACP, and ALP expression. Enzyme-linked immunosorbent assays further confirmed the qPCR results. Tartrate-resistant acid phosphatase staining showed that HFY15 slowed retinoic acid-induced osteoclast formation. Microcomputed tomography showed that HFY15 reduced trabecular separation and increased the percent bone volume, trabecular numbers, trabecular thickness, and bone mineral density in the rats in vivo. These findings indicate that HFY15 may help prevent retinoic acid-induced secondary osteoporosis in vivo.

## 1. Introduction

Secondary osteoporosis (OP) is caused by certain diseases and treatments that interfere with bone density and cause bone loss [1]. Up to 30% of postmenopausal women and 50% of men with osteoporosis may have an underlying cause [2]. People with osteoporosis generally do not experience symptoms. Osteoporosis often goes undetected for many years and is not diagnosed until a person breaks a bone. Common bone fractures related to osteoporosis include fractures of the hip, wrist, or spine. Osteoporosis occasionally causes symptoms [3]. The underlying pathogenesis of secondary osteoporosis is usually multifactorial. Properly treating the causes of osteoporosis can reduce the risk of fractures and prevent unnecessary treatment with anti-reabsorption drugs [4, 5].

Many diseases, drugs, and lifestyle factors can cause secondary osteoporosis. Common medical disorders that contribute to secondary osteoporosis are cancers that cause bone loss, including bone, breast, and prostate cancers, as well as hormonal imbalances such as hyperthyroidism, a condition that causes excessive thyroid function [6]. Diseases such as kidney failure [7], rheumatoid arthritis [8], systemic lupus erythematosus [9], Sjogren's syndrome [10], dermatomyositis [11], and mixed connective tissue disease [12] can also cause secondary osteoporosis. Long-term treatment with glucocorticoids can lead to decreased intestinal calcium absorption, increased urinary calcium excretion, and increased serum thyroid hormones, leading to bone loss [13, 14]. Prolonged use of drugs such as proton-pump inhibitors [15], antiepileptic drugs [16], diuretics [17], anticoagulants [18], and cyclosporine A [19] can cause severe bone loss. In addition to diseases and drug use, poor living habits, such as alcoholism, smoking, low physical activity, and long-term lack of vitamin intake, can lead to bone loss, which can in turn cause secondary osteoporosis [20].

In the treatment of secondary osteoporosis, changing living habits and increasing the absorption of vitamins and calcium are conventional methods. In addition, bisphosphonates, calcitonin, estrogen, and estrogen receptor modulators are also commonly used in the clinical treatment of osteoporosis and could increase the bone density of patients. However, these drugs can have safety, tolerability, and other issues and can bring huge economic burdens to patients. In addition, conventional bone loss treatments are not always effective. Therefore, new approaches to increase bone density are needed.

The role of the intestinal microbiota in regulating human health and disease is receiving increasing attention [21, 22]. Reports have indicated that prebiotics could increase bone density especially in combination with probiotics [23]. Probiotics have also been shown to increase cortical bone thickness in chicken [24] and reduce bone loss in aging mice [25] and improve bone density in male mice [26]. Some other studies also have shown that intestinal colonization of probiotics in mice can affect bone formation and remodeling and may help prevent OP [27, 28]. While the intestine is known to be key for calcium and vitamin D metabolism, the effect of probiotics community changes on bone health, especially in secondary osteoporosis, has not been thoroughly examined.

*Lactobacillus plantarum* HFY15 (HFY15) is a lactic acid bacterium that our research team isolated and identified from natural yak yogurt. In this study, we used retinoic acid to establish a rat model of secondary OP and investigated the preventive effect of HFY15 on OP. We found that HFY15 supplementation increased the expression of osteogenic marker genes and stimulated bone formation. The results may provide a new and efficient strategy for preventing and treating OP.

## 2. Materials and Methods

### 2.1. Laboratory Strain

*Lactobacillus plantarum* HFY15 was isolated from yak yogurt in Sichuan Province, China, and is preserved in the China General Microbiological Culture Collection Center (CGMCC No. 16648), Beijing, China. 1 ml of reconstituted yogurt sample was taken, put in 9 ml of sterile saline, mixed thoroughly, and diluted gradually. 100 *μ*L of 10^−4^, 10^−5^, 10^−6^, and 10^−7^ dilutions were taken and applied to MRS (DeMan-Rogosa-Sharpe) agar plates. The colony morphology was observed after incubation at 37°C for 24–48 hours. Colonies with similar morphology were selected. Pure colonies were inoculated with MRS liquid medium (5 mL) and cultured at 37°C for 18–24 hours. The above 1 mL medium was centrifuged at 12000 rpm for 1 minute, the supernatant was discarded, 200–500 *μ*L of sterile normal saline was added, and detection was done under a microscope after Gram staining. The purified target strain was reinoculated into MRS liquid medium (5 mL). After 18–24 hours at 37°C, DNA was extracted (Dynabeads TM DNA DIRECT TM Universal Kit, Thermo Scientific, Wilmington, DE, USA). The 16S rDNA gene of lactic acid bacteria was amplified by PCR, and the product was checked by agarose gel electrophoresis. Finally, the identified correct strains are made into a bacterial suspension for subsequent experiments.

### 2.2. In Vivo Rat Model of Osteoporosis

Forty 8-week-old female Wistar rats (weighing 250–300 g each) were obtained from the Laboratory Animal Center of Chongqing Medical University, Chongqing, China, and used as experimental animals. The rats were randomly housed at 2 per cage, maintained on a 12 : 12 hr light:dark cycle, and given access to food and water ad libitum. The rats were randomly divided into the control, model, zoledronic acid (ZA), and HFY15 groups (*n* = 10 per group). The rats were acclimated to laboratory conditions for one week before initiating prophylactic treatment. Rats in the control and model groups were intragastrically administered 1 mL of saline per 100 g body weight daily for 2 weeks. Rats in the zoledronic acid and HFY15 groups were intragastrically administered 1 mL of 10^10^ CFU/kg of HFY15 per 100 g body weight daily for 2 weeks. All groups except the control group also received retinoic acid (80 mg/kg/d) intragastrically starting on day 14 for 4 weeks. The control group received an equal volume of saline. During these four weeks, the HFY15 group continued receiving the original bacterial dose daily, while rats in the zoledronic acid group were injected via tail vein with one dose of 0.5 mL per 100 g body weight of zoledronic acid (Chiatai Tianqing, Jiangsu, China). After four weeks, all rats fasted for 24 h and then were humanely killed by cervical dislocation. Sera and spinal cords were collected from the rats and stored at −80°C until further use. The tibias and femurs were collected and fixed in 10% (v/v) buffered formaldehyde for histological analysis and microcomputed tomography (micro-CT).

### 2.3. Determination of Serum Biochemical Indicators and Cytokines

Blood from the rats was collected and centrifuged at 4000 rpm for 10 min, and then the supernatant (serum) was collected. Serum calcium and phosphorus levels were determined following the kit's instructions (Nanjing Jiancheng Bioengineering Institute, Nanjing, Jiangsu, China). Cytokines levels were assayed using rat bone-specific alkaline phosphatase (BAP, ml037086), rat osteocalcin (BGP, ml002883), rat insulin-like growth factor-1 receptor (IGF-1R, ml059459), rat anti-tartaric acid phosphatase 5b (TRACP-5b, ml003177), and rat gamma aminobutyrate transaminase (GABA, ml064273) cytokine assay kits (Shanghai Enzyme-linked Biotechnology Co., Ltd., Shanghai, China).

### 2.4. RNA Extraction, Reverse Transcription, and Real-Time qPCR

Total RNA was extracted from the spinal cords using TRIzol reagent and the Ultrapure RNA Kit (Invitrogen, Carlsbad, CA, USA). RNA concentrations were determined using a Nanodrop 1000 (Thermo Scientific). For real-time quantitative reverse transcription (qRT-PCR), the first-strand cDNA was synthesized using a RevertAid First-Strand cDNA Synthesis Kit (Thermo Scientific). The system was reacted at 95°C for 60 s, followed by 40 cycles at 95°C for 30 s, and annealing at 72°C for 30 s. Finally, the DNA was detected at 95°C for 30 s and 5°C for 35 s. The 2^−ΔΔ*Ct*^ method was used to determine the relative gene expression levels.  Table 1 lists the sequences of the PCR primers used.

### 2.5. Histological Observations

After the rats were sacrificed, their tibias and femurs were immediately removed and fixed in 10% (v/v) buffered formaldehyde. The proximal tibial femurs were routinely processed, embedded in paraffin, sectioned, stained with tartrate-resistant acid phosphatase (TRAP), and observed under a BX43 microscope (Olympus, Tokyo, Japan). Osteoclasts with three or more nuclei were counted in six randomly selected fields, and the average was calculated to represent the number of osteoclasts in each histological section [29].

### 2.6. Bone Imaging by Micro-CT

Fixed femurs were scanned using a Bruker Micro-CT Skyscan 1272 system (Kontich, Belgium) at a 20 *μ*m voxel resolution obtained from 720 views. The beam angle of increment was 0.5°; the beam strength was set at 80 peak kV and 450 μA. Each run consisted of bones from the control, model, zoledronic acid, and HFY15 groups, and a calibration phantom was used to standardize the grayscale values and maintain consistency. Based on the autothreshold and isosurface analyses of multiple bone samples, a fixed threshold (760) was used to separate the bone from the bone marrow. Bone measurements were blinded; thus, the experimental condition from which the analyzed bone was taken was unknown until the final data were pooled. Tissue volume, bone volume, trabecular number, trabecular thickness, trabecular separation, and bone mineral density were determined using a Bruker MicroCT Skyscan 1272 system software application to visualize and analyze the volumetric imaging data [30].

### 2.7. Statistical Analysis

Data are expressed as the mean ± standard deviation. SPSS software, version 22 (IBM Corporation, North Castle, NY, USA) was used for the analysis of variance with the post hoc Duncan's new multiple range test. Differences at *p* < 0.05 were considered statistically significant. All figures were drawn using Origin 8.0 software [31].

## 3. Results

### 3.1. Serum Levels of Biochemical Indicators and Cytokines

Table 2 presents the calcium and phosphorus levels in the rat serum. Levels in the model group were the lowest, and those in the zoledronic acid and HFY15 groups were significantly higher than those in the control and model groups. Serum cytokine detection assays showed that serum levels of BAP, BGP, IGF-1R, and GABA were the lowest in the model rats and were significantly higher in the zoledronic acid and HFY15 groups than those in the control and model groups. Serum levels of TRACP-5b were the highest in the model group and significantly lower in the zoledronic acid and HFY15 groups than those in the control and model groups.

### 3.2. Expression of Wnt/*β*-Catenin Signaling Pathway Genes in the Spinal Cord

**Figure 1:**
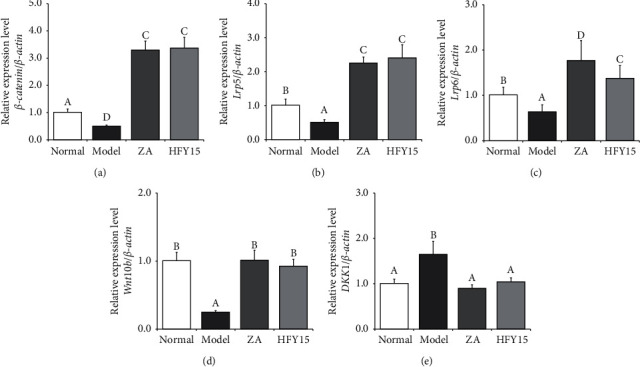
*β-Catenin*, *Lrp5*, *Lrp6*, *Wnt10b,* and *DKK1* expression in the spinal cord of rats. Different letters over the bars within each panel indicate a significant difference between the groups (*P* < 0.05). (a) *β*-Catenin, a Wnt signaling pathway key protein. (b) Lrp5, low-density lipoprotein receptor-related protein 5. (c) Lrp6, low-density lipoprotein receptor-related protein 6. (d) Wnt10b, Wnt signaling pathway key protein. (e) DKK1, dickkopf-related protein 1.

The classic Wnt/*β*-catenin pathway plays an important role in osteoblast differentiation and proliferation. Studies have shown that factors that regulate any of the classic Wnt/*β*-catenin signaling pathways can affect osteoblast differentiation and proliferation. In the Wnt/*β*-catenin signaling pathway, *β*-catenin [32], Lrp5/6 [33], and Wnt10b [34] positively regulate the bone formation, while DKK1 [35] negatively regulates bone formation by inhibiting the pathway. *β-Catenin*, *Wnt10b*, *Lrp5,* and *Lrp6* mRNA expression levels were the lowest in the spinal cords of the model rats and the highest in the spinal cords of the zoledronic acid and HFY15 groups. *DKK1* expression levels were the highest in the model group and the lowest in the zoledronic acid and HFY15 groups (Figure 1).

### 3.3. Expression of OPG/RANK/RANKL Signaling Pathway Genes in the Spinal Cord

**Figure 2:**
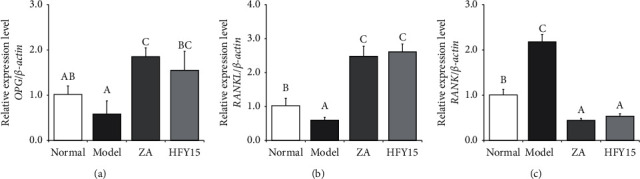
*OPG*, *RANKL* and *RANK* expression in the spinal cord of rats. Different letters over the bars within each panel indicate a significant difference between the groups (*P* < 0.05). (a) OPG, osteoprotegerin. (b) RANKL, receptor activator of the nuclear factor-*κ*B ligand. (c) RANK, receptor activator of nuclear factor-*κ*B.

The osteoprotective protein (OPG)/nuclear factor-K*β* receptor-activating factor (RANK)/nuclear factor-K*β* receptor-activating factor ligand (RANKL) signaling pathway regulates osteoclast function during bone reconstruction [36,37]. Cells release RANKL, which combines with RANK on the surfaces of osteoclasts to promote osteoclast differentiation and activation through the NF-*κ*B, JNK, and protein kinase B pathways. OPG can competitively inhibit the binding of RANK and RANKL to promote bone cell function and reduce bone destruction. mRNA expression levels of *OPG* and *RANKL* were the lowest in the spinal cords of the model group and the highest in the spinal cords of the zoledronic acid and HFY15 groups. *RANK* expression levels were the highest in the model group and the lowest in the zoledronic acid and HFY15 groups (Figure 2).

### 3.4. Expression of Osteogenesis and Osteogenesis Marker Genes in the Spinal Cord

**Figure 3:**
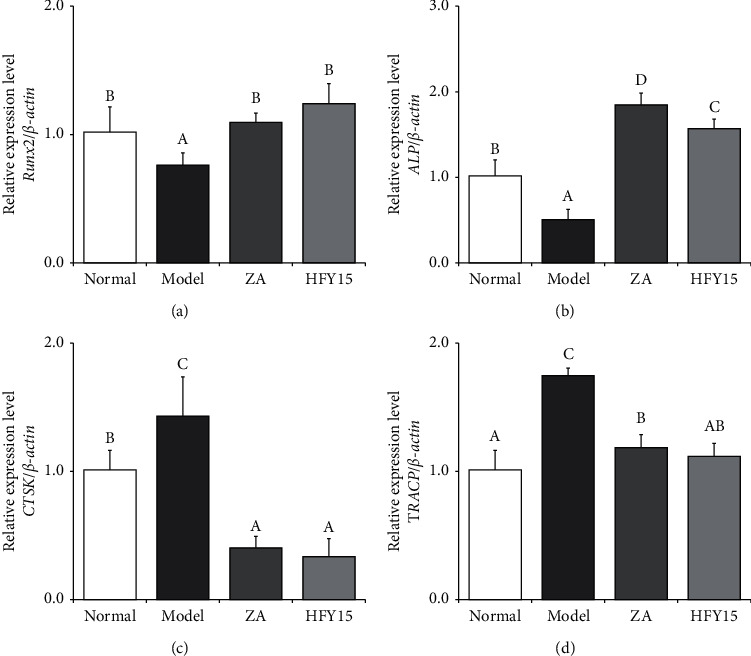
*Runx2*, *ALP*, *CTSK,* and *TRACP* expression in the spinal cord of rats. Different letters over the bars within each panel indicate a significant difference between the groups (*P* < 0.05). (a) Runx2, runt-related transcription factor 2. (b) ALP, alkaline phosphatase. (c) CTSK, cathepsin K. (d) TRACP, tartrate-resistant acid phosphatase.

mRNA expression levels of the osteogenesis marker gene *Runx2* were the lowest in the spinal cords of the model group and the highest in the spinal cords of the zoledronic acid and HFY15 groups. Expression levels of alkaline phosphatase (*ALP*) were the lowest in the model group and the highest in the zoledronic acid and HFY15 groups. Expression levels of the osteogenesis marker genes *CTSK* and *TRACP* were the highest in the model group and the lowest in the zoledronic acid and HFY15 groups. HFY15 strongly affected the bone marker gene expression in the spinal cords of the HFY15 group; these expression levels were near those of the zoledronic acid and control groups (Figure 3).

### 3.5. Pathological Observation of the Femurs and Tibias

**Figure 4:**
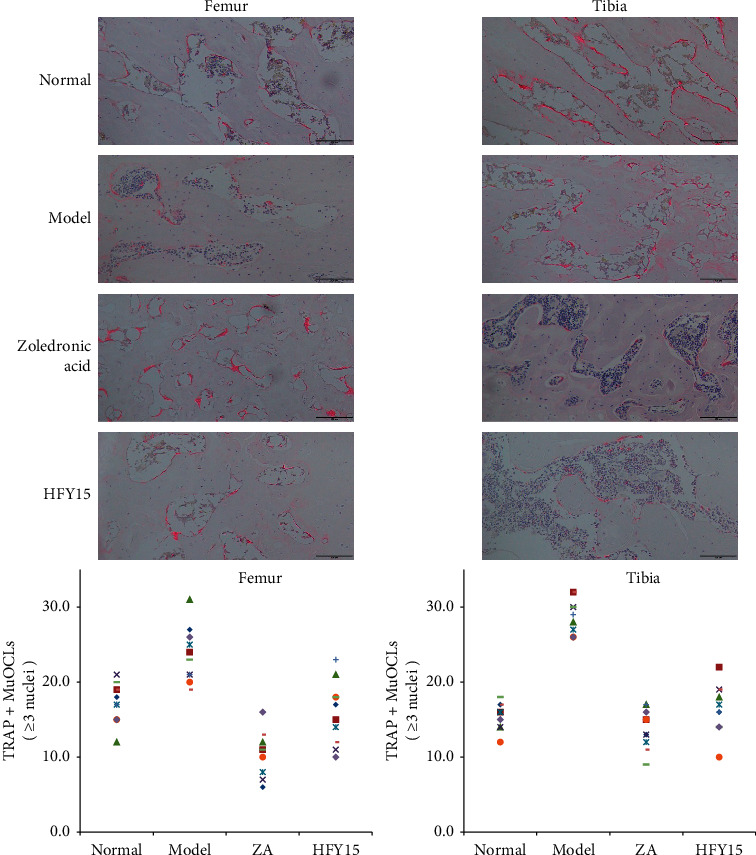
Pathological observation of rats' femur and tibia stained with TRAP. Magnification: 100×. Normal: normal rats control; Model: rats treated with retinoic acid (80 mg/kg/d); Zoledronic Acid: rats treated with retinoic acid (80 mg/kg/d) and a single dose of 0.5 mL/100 g zoledronic acid; HFY15: rats treated with retinoic acid (80 mg/kg/d) and 1 mL/100 g with 10^10^ CFU/kg of *Lactobacillus plantarum* HFY15.

Figure 4 shows histological micrographs of the rat femurs and tibias. Osteoclast numbers, morphology, and degree of fusion were normal in the femurs and tibias of the control group. The number of osteoclasts in the model group increased significantly, with many fused giant multinucleated cells. In contrast, osteoclast numbers in the zoledronic acid and HFY15 groups were reduced to the level of the control group.

### 3.6. Micro-CT of the Femurs and Tibias

**Figure 5:**
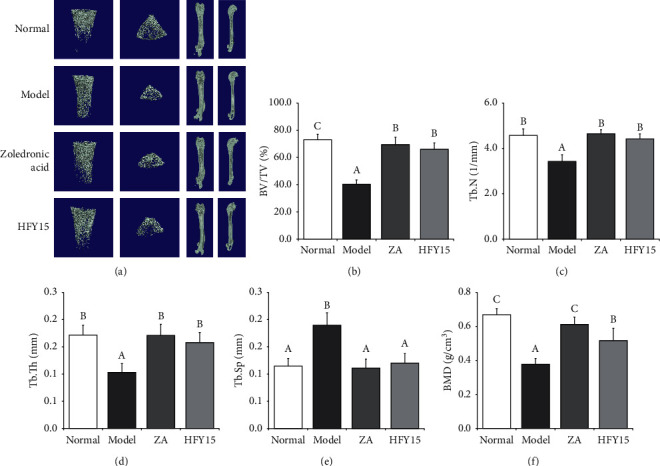
Micro-CT results of rats' femur. All images are representative of the respective groups. Normal: normal rats control; Model: rats treated with retinoic acid (80 mg/kg/d); Zoledronic Acid: rats treated with retinoic acid (80 mg/kg/d) and a single dose of 0.5 mL/100 g zoledronic acid; HFY15: rats treated with retinoic acid (80 mg/kg/d) and 1 mL/100 g with 10^10^ CFU/kg of *Lactobacillus plantarum* HFY15.

Figure 5 shows the micro-CT results for the rat femurs. In the control group, the femurs had normal values for percent bone volume (BV/TV), trabecular number (Tb.N), trabecular thickness (Tb.Th), trabecular separation (Tb.Sp), and bone mineral density (BMD). BV/TV, Tb.N, Tb.Th, and BMD were the lowest in the model group femurs, while Tb.Sp was the highest in the model group. The femurs of the zoledronic acid and HFY15 groups were similar to those of the control group; BV/TV, Tb.N, Tb.Th, and BMD were significantly increased, while Tb.Sp was significantly decreased.

## 4. Discussion

All-trans retinoic acid is an active metabolite of vitamin A in the retinoid family. Retinoids, through their cognate nuclear receptors, exert potent effects on cell growth, differentiation, and apoptosis and have potential applications in cancer therapy and chemoprevention [38]. Retinoic acid also plays an important role in maintaining immune homeostasis [39] and treating Alzheimer's disease [40]. Long-term use of retinoic acid can cause adverse effects such as liver damage, bone loss, and chapped skin. Many studies have used retinoic acid to construct animal models of secondary osteoporosis [41–44]. Therefore, we treated rats with retinoic acid to create a secondary OP model.

Like other body tissues, bone constantly undergoes cellular metabolism, or bone metabolism, which is divided into two stages: formation and remodeling. Bone formation plays a major role in individual growth and development, while bone remodeling continues throughout the life cycle [45, 46]. Osteoblast differentiation into osteocytes is a complex process involving multiple signaling pathways, of which, the Wnt/*β*-catenin and OPG/RANK/RANKL signaling pathways are the most well studied. Studies have shown that genes involved in the Wnt/*β*-catenin and OPG/RANK/RANKL signaling pathways exert important effects on osteogenesis [47–50]. After modeling, we directly detected the related gene expressions to explore the effect of HFY15 on bone mass. qPCR analysis showed that the expression levels of *β-catenin*, *Wnt10b*, *Lrp5*, *Lrp6*, *OPG*, *RANKL,* and *Runx2* were the lowest in the model group, while the expressions of the negatively regulated genes, *DKK1*, *ALP*, *CTSK,* and *TRACP*, increased, indicating successful construction of our model of secondary osteoporosis. Similarly, the analysis of the qPCR results showed that HFY15 positively regulated bone formation in the rats, and the effect was similar to that of zoledronic acid. Many genes are involved in these two pathways, and we did not further verify the changes in gene expression levels via western blot. However, enzyme-linked immunosorbent assay (ELISA) results revealed changes in the IGF-1R content and BAP, BGP, and TRACP expression levels, which confirmed the qPCR results.

A small amount of retinoic acid can promote bone formation, but a large amount of retinoic acid intake can damage rat ovaries, cause decreased estrogen secretion, weaken the inhibitory effect on osteoclasts, and increase the activity and number of osteoclasts [51, 52]. During bone remodeling, osteoclasts derived from mononuclear macrophages fuse into multinucleated osteoclasts, which absorb bone and reduce bone mass [53–55]. Counting the osteoclasts with three or more nuclei can accurately reflect the occurrence of bone remodeling. We stained bone tissue sections with TRAP, which turned the cytoplasm in the osteoclasts burgundy and the nuclei light blue. After sectioning and staining the bone tissue, we counted the multinucleated osteoclasts from 40 rats. The model group had the most multinucleated osteoclasts, while the treatment and HFY15 groups had fewer osteoclasts. This suggests that HFY15 can effectively prevent retinoic acid from promoting bone resorption. Because rat bone tissue is large, it is difficult to count the total number of multinucleated osteoclasts in one section and could induce error. Therefore, we randomly selected three fields of view from each slice and averaged the counts from each view to represent the number of multinucleated osteoclasts from each section.

qPCR, ELISA, and histological micrographs can reflect only the localization of osteogenesis and bone resorption and cannot directly or effectively represent the real changes in bone mass. Therefore, a technology that can directly respond to changes in bone mass must be used to accurately detect a bone mass. Micro-CT is a noninvasive technology that combines imaging and high-resolution histological detection. The difference in the X-ray attenuation coefficient between bone and other body tissues allows using micro-CT for bone imaging to directly indicate bone quality [56, 57]. We randomly selected 6 femurs and 6 tibias from the four groups for a CT scan to reflect the real situation of the bone mass. The BV/TV, Tb.N, Tb.Th, and BMD were the lowest, while Tb.Sp was the highest in the model group, indicating that our model was successfully established. These values returned to normal in the femurs of the rats in the zoledronic acid and HFY15 groups, again indicating that the HFY15 lactobacillus strain isolated and purified in our laboratory exerted a preventive effect on secondary OP, and this effect was similar to that of zoledronic acid.

Probiotics are a group of active microorganisms that benefit the host by colonizing in the body and changing the composition of part of the host's flora. Adjusting the host's mucosal and immune system functions or adjusting the intestinal flora balance promotes nutrient absorption and maintains intestinal tract health, thereby producing single or mixed microorganisms with well-defined effects that benefit host health [58–60]. Before researching the effects of probiotics on life activities, researchers must ensure that probiotics can colonize the intestinal tract and resist damage from gastric acid and bile salts [61]. Our research group performed relevant experiments on strain HFY15 after isolating and purifying it. After treatment with artificial gastric acid and bile salt, HFY15 survival rates were 86.26 ± 10.78% and 53.45 ± 2.74%, respectively. Before starting the experiment, we administered HFY15 to the rats for two weeks to ensure HFY15 colonization in the rats.

Our results showed that HFY15 had a good preventive effect on retinoic acid-induced osteoporosis, but the specific mechanism is unclear. Whether HFY15 alone affects the intestinal absorption of calcium and phosphorus or the balance of the intestinal flora, which in turn affects bone formation and absorption, is uncertain. How HFY15 participates in the Wnt/*β*-catenin and OPG/RANK/RANKL signaling pathways as well as the key factors involved requires further study. Our follow-up research will address these questions. Professor Roberto Pacifici's research on the effects of *Lactobacillus rhamnosus* (LGG) on bone mass in mice and rabbits showed that LGG can increase butyrate levels in the intestines. In turn, butyrate promotes T cells in the bone marrow to produce Wnt10b, which is essential for bone growth [62]. This result inspired our research, and our ELISA results showed that HFY15 positively regulated GABA. This route will be the focus of our subsequent research.

## 5. Conclusion

This study investigated the preventive effects of *Lactobacillus plantarum* HFY15 on secondary OP in rats and showed that this strain effectively improved the expression of osteogenic marker genes in rat serum and spinal cords and promoted bone formation in rats in vivo. This study built a foundation for further research on HFY15. Because only animal experiments were conducted in this study, human clinical trials are needed to confirm the preventive effects of HFY15 on the secondary OP.

## Figures and Tables

**Table 1:** Sequences of primers used in this study.

Gene name	Sequence
*β-actin*	Forward: 5′- TCAGGTCATCACTATCGGCAAT -3′
	Reverse: 5′- AAAGAAAGGGTGTAAAACGCA -3′
*β-catenin*	Forward: 5′- GGTGAAAATGCTTGGGTCGC -3′
	Reverse: 5′- AGATCTGAAGGCAGTCTGTCGTAA -3′
*Wnt10b*	Forward: 5′- GTGGGAATGGGGTGGCTGTA -3′
	Reverse: 5′- CCGCATTCTCGCCTGGAT -3′
*Lrp5*	Forward: 5′- GCATCATCCTGTCCCTCTTCG -3′
	Reverse: 5′- GACCGTGCTGTGAGCCACC -3′
*Lrp6*	Forward: 5′- ACAGACTGGAGCCGACGCA -3′
	Reverse: 5′- GCCAAGCAAAGGTGGGAGC -3′
*Runx2*	Forward: 5′- GAACCAAGAAGGCACAGACAGAA -3′
	Reverse: 5′-GGCGGGACACCTACTCTCATACT -3′
*ALP*	Forward: 5′- GCGACAGCAAGCCCAAGAG -3′
	Reverse: 5′- CTCCAGCCGTGTCTCCTCG -3′
*RANKL*	Forward: 5′- TGGAGAGCGAAGACACAGAAGC -3′
	Reverse: 5′- GGTGAGGTGAGCAAACGGC -3′
*OPG*	Forward: 5′- AATTGGCTGAGTGTTCTGGTGG -3′
	Reverse: 5′- GCTGGAAAGTTTGCTCTTGCG -3′
*DKK1*	Forward: 5′- GGCTCTGTCTGCCTCCGATC -3′
	Reverse: 5′- GCCTTTCCTCCTGTGCTTGG -3′
*RANK*	Forward: 5′- GGCTTCTTCTCAGATGTCTTTTCG -3′
	Reverse: 5′- TGATTCCGTCGTCCCTTGGT -3′
*TRACP*	Forward: 5′- GTGGCTGTGGGTGACTGGG -3′
	Reverse: 5′- CAAAGGTCTCCTGGAACCTCTTG -3′
*CTSK*	Forward: 5′- AAGGCAGCTAAGTGCAGAGGG -3′
	Reverse: 5′- GGTTCACATTATCACGGTCGC -3′

**Table 2:** Levels of biochemical indicators and cytokines in rats' serum.

Group	Normal	Model	Zoledronic acid	HFY15
Calcium (mmol/L)	1.30 ± 0.11^b^	1.08 ± 0.20^a^	1.16 ± 0.14^ab^	1.50 ± 0.23^c^
Phosphorus (mmol/L)	1.95 ± 0.19^b^	1.65 ± 0.19^a^	2.38 ± 0.32^c^	2.38 ± 0.33^c^
BAP (ng/ml)	11.21 ± 0.90^b^	9.86 ± 1.52^a^	12.66 ± 0.98^c^	13.81 ± 1.75^c^
BGP (ng/ml)	1.96 ± 0.76^b^	1.31 ± 0.39^a^	3.26 ± 1.12^c^	3.32 ± 1.68^c^
IGF-1R (pg/ml)	615.32 ± 115.61^b^	320.93 ± 101.72^a^	555.48 ± 222.39^b^	535.71 ± 259.27^b^
TRACP-5b (ng/ml)	0.62 ± 0.36^ab^	2.61 ± 0.56^c^	0.30 ± 0.20^a^	0.70 ± 0.33^b^
GABA (umol/L)	7.49 ± 0.31^b^	7.10 ± 0.40^a^	7.43 ± 0.24^b^	7.89 ± 0.39^c^

Values presented are the mean ± SD (*n* = 10/group). Mean values with different superscript letters in the same row are significantly different (*P* < 0.05). Normal: normal rats control; Model: rats treated with retinoic acid (80 mg/kg/d); Zoledronic Acid: rats treated with retinoic acid (80 mg/kg/d) and single treatment with 0.5 mL/100 g zoledronic acid; HFY15: rats treated with retinoic acid (80 mg/kg/d) and 1 mL/100 g with 10^10^ CFU/kg of *Lactobacillus plantarum* HFY15. BAP: bone-specific alkaline phosphatase. BGP: osteocalcin. IGF-1R: insulin-like growth factor-1 receptor. TRACP-5b: tartrate-resistant acid phosphatase 5b. GABA: *γ*-aminobutyric acid.

## Data Availability

No data were used to support this study.
